# Legionnaires’ Disease Outbreak in Murcia, Spain

**DOI:** 10.3201/eid0908.030337

**Published:** 2003-08

**Authors:** Ana García-Fulgueiras, Carmen Navarro, Daniel Fenoll, José García, Paulino González-Diego, Teresa Jiménez-Buñuales, Miguel Rodriguez, Rosa Lopez, Francisco Pacheco, Joaquín Ruiz, Manuel Segovia, Beatriz Baladrón, Carmen Pelaz

**Affiliations:** * Regional Health Council of Murcia, Murcia, Spain; † Council of Murcia, Murcia, Spain; ‡ Virgen de la Arrixaca Hospital, Murcia, Spain; § Morales Meseguer Hospital, Murcia, Spain; ¶ Health Institute of Carlos III, Madrid, Spain

**Keywords:** Legionnaires’ disease, community outbreak, case-control study, environmental exposure

## Abstract

An explosive outbreak of Legionnaires’ disease occurred in Murcia, Spain, in July 2001. More than 800 suspected cases were reported; 449 of these cases were confirmed, which made this the world’s largest outbreak of the disease reported to date. Dates of onset for confirmed cases ranged from June 26 to July 19 , with a case-fatality rate of 1%. The epidemic curve and geographic pattern from the 600 completed epidemiologic questionnaires indicated an outdoor point-source exposure in the northern part of the city. A case-control study matching 85 patients living outside the city of Murcia with two controls each was undertaken to identify the outbreak source; the epidemiologic investigation implicated the cooling towers at a city hospital. An environmental isolate from these towers with an identical molecular pattern as the clinical isolates was subsequently identified and supported that epidemiologic conclusion.

Legionnaires’ disease (LD) has been an emergent disease since the 1970s. In the last few years, the increased use of a simple test for detecting urinary antigen *Legionella* *pneumophila* serogroup 1 in patients with pneumonia has facilitated diagnosis (*1*). Transmission by aerosols has been extensively reported, and evidence of *Legionella* in aerosols derived from cooling towers has been provided (*2*–*5*). Although a considerable body of epidemiologic evidence exists for the association of LD outbreaks with aerosols produced by cooling towers, some controversy exists about the role that cooling towers play in LD (*6*–*13*).

We describe an explosive outbreak of LD that occurred in July 2001 in Murcia, a municipality with 360,000 inhabitants in southeastern Spain. We also report results of a case-control study performed to identify the source of this outbreak, which turned out to be a cooling tower. The outbreak of pneumonia was first detected on July 7. At the end of the first day of active surveillance, July 8, approximately 100 cumulative suspected cases were reported. More than 800 suspected cases were recorded by July 22, when the last case was treated, 2 weeks after the onset of the investigation. The epidemiologic investigation using a case-control study emphasizes a combination of strategies to measure and analyze an outbreak of LD that occurs in an area with many large potential sources of environmental contamination.

## Methods

### Case Detection

An active surveillance system to detect patients with any form of pneumonia was established on July 8 at all hospitals in the region of Murcia. Any reported case of pneumonia was considered a suspected case of LD if this diagnosis could not be ruled out. A confirmed case of LD was defined as a case of pneumonia with laboratory evidence of acute infection with *Legionella* including a) isolation of any species or serogroup of *Legionella* from respiratory secretions, lung tissue, or blood, b) a fourfold or higher rise in antibody titers from 1:128 against *L. pneumophila* SG1 by immunofluorescence or microaglutination in paired acute- and convalescent-phase serum specimens, or c) detection of *L. pneumophila* antigen in urine.

An epidemiologic questionnaire to elicit information on clinical aspects, predisposing factors, risk factors, place of residence, and recent urban mobility within the city of Murcia was administered to 662 persons with suspected cases, most within 24 to 48 hours after the case was reported. A computerized database was set up as well as maps showing geographically referenced cases and a spatial analysis by census division that used a geographic information system (*14*).

### Case-Control Study

Inclusion in the study was restricted to patients who had confirmed LD, were residents outside the city of Murcia, and had been reported July 8–20 as case-patients. Each case-patient was matched to two controls according to place of residence, sex, and age. Controls were randomly selected from the population of the same area of residence and health district as the matched patient.

A standardized questionnaire to interview patients and controls was designed. It focused on urban mobility and exposure to outside air within the northern part of Murcia 2 weeks before the patient’s onset of illness. Patients and controls were interviewed in person at home between July 25 and August 8. Itineraries of all participants, including information about means of transport and frequency of trips, were outlined on a map of Murcia. In addition, any travel into or visit to 30 specific zones of the city in which putative sources of contaminated aerosols were located was recorded. The questionnaire also requested information about place of residence and work, occupation, education level, employment status, smoking habit, alcohol intake, chronic lung disease, diabetes, renal or heart disease, malignancy, immunocompromising disease, organ transplant, therapy with corticosteroids, and other risk factors for LD within 2 weeks before illness.

A multivariate analysis that used conditional logistic regression was conducted to calculate odds ratios (OR) with 95% confidence intervals (CI) as estimates of the relative risk for LD associated with a person’s travel through each zone; we controlled for the confounding effects of traveling through other zones. Any zone of exposure that was significant in univariate analysis or showed biologic plausibility as a source was entered into the multiple analysis. The frequency with which participants visited Murcia city was also introduced into the multivariate analysis. Statistical analysis was conducted with STATA software (*15*).

### Strategies of Analysis

Exposure zones were analyzed in two ways after codification of the information obtained from each patient or control as he or she traveled or did not travel through a) the area defined by the block around a building with a cooling tower or the block around an ornamental fountain (in this way, 30 zones of the northern part of the city were coded), and b) the area delineated by a circle of 200 m radius around a cooling tower or a large ornamental fountain. Therefore, eight high-risk zones were studied.

In all cases, for each area of exposure, how the patient or control traveled through the area (i.e., walking [a category that also included bicycling or motorbiking] or driving [a car, bus, or truck]) was specified. This information was analyzed for the following: a) walking versus not passing through a zone or b) walking versus not passing through an area or traveling through it by car. Finally, for all possibilities, data were analyzed in two further ways: a) complete, which took into account all persons in the study, or b) restricted, which took into account only the trios of case-patients and their two paired controls in which all three persons stated that they had visited Murcia in the study period.

### Environmental Investigation

Possible sources of aerosols were inspected, and water samples were collected from the water supply network and from 339 installations (e.g., cooling towers, storage tanks, and decorative fountains). Cooling towers were identified by aerial inspection because no census of these installations was available. Attack rates by residence were used to determine in which locations inspections and environmental samplings could be conducted.

### Microbiologic Study

Environmental samples were processed according to ISO 11731/1998. Environmental and clinical *L. pneumophila* serogroup 1 isolates were typed by monoclonal antibody (MAb) with International and Dresden MAb panels (*16*,*17*) and compared by three molecular methods, amplified fragment length polymorphism (AFLP), pulsed-field gel electrophoresis (PFGE)-*Sfi*I, and arbitrarily primed–polymerase chain reaction (AP-PCR) (*18*–*20*).

## Results

### Descriptive Epidemiology

The outbreak of pneumonia was detected on the evening of July 7, when the Regional Department was notified that an increase in pneumonia cases had occurred in three hospitals in Murcia. *Legionella* antigen was detected in some patients’ urine. Approximately 800 suspected cases were reported July 8–22; confirmed cases numbered 449. We estimate that 636–696 persons were affected. These estimates took into account the sensitivity of the antigen test in urine of 70% (*4*) and the background number of pneumonia cases estimated from the median of patients admitted with pneumonia in the region’s hospitals during the summers from 1996 through 2000.

**Figure 1:**
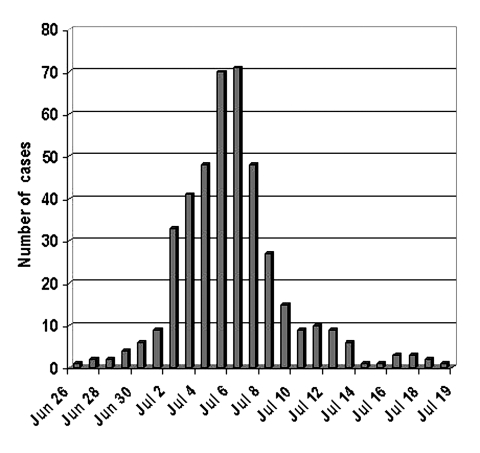
Confirmed cases of Legionnaires’ disease by date of onset of illness, Murcia, Spain, June 26–July 19, 2001.

Onset of illness of the first confirmed case-patient was June 26. Until July 1, only a small number of cases occurred. After this date, the outbreak became explosive, with most cases occurring in <10 days (83% of confirmed cases had onset from July 2 through July 9). The last case-patient became ill on July 19 (Figure 1). The outbreak was considered compatible with massive exposure to a common source of contamination. When LD’s incubation period was taken into account, the maximum emission was estimated to have occurred June 29–July 1 and to have ended completely July 9–17.

**Figure 2:**
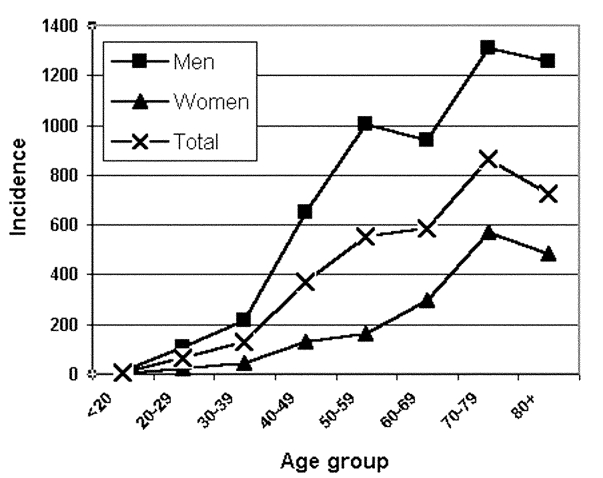
Confirmed cases of Legionnaires’ disease within the city of Murcia, Spain. Specific incidence rates by sex and age (per 100,000).

Hospital admission was necessary for 64% of all reported case-patients and 74% of confirmed case-patients. Six deaths from LD were confirmed to be directly related to this outbreak, five confirmed cases and one suspected. Therefore, the case-fatality rate was 1.1% for confirmed cases only and 0.9% for total estimated cases. For all confirmed case-patients, 74% were men and 26% women. The age range was from 19 to 91 years; 70% were >50, and 29% were >70. The incidence rate increased with age in both sexes and was higher in men in all age groups (Figure 2).

**Figure 3:**
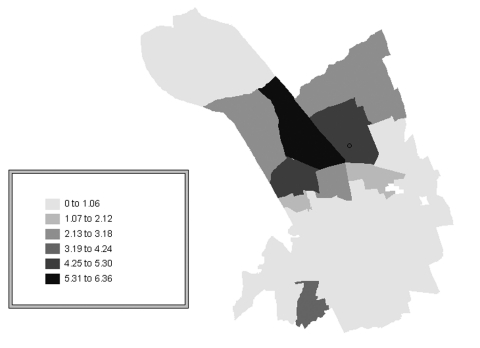
Confirmed cases of Legionnaires’ disease within Murcia city, Spain. Standardized Incidence Ratio (SIR) by neighborhood.

Of the confirmed case-patients, 68% lived in Murcia city proper, 16% in the satellite districts within the municipality of Murcia, and 16% in other municipalities of the region. To evaluate the risk by quarters within the city of Murcia, the Standardized Incidence Ratio (SIR) was used. Three neighborhoods located in the northern part of the city had the highest incidence rate (4.9–6.7 per 1,000 population), significantly higher than the average for the city of Murcia (Table 1, Figure 3). According to epidemiologic interviews, 95% of the confirmed case-patients lived, worked, or visited in the northern districts in the 10 days before the outbreak began.

**Table 1:** Confirmed cases of Legionnaires’ disease, Murcia, Spain

Neighborhood	Confirmed cases	Inhabitants	Incidence per 1,000	SIR^a^ (95% CI)
Sta. Mª de Gracia	90	13,410	6.7	6.3 (5.1 to 7.8)
Vistalegre	62	12,677	4.9	4.8 (3.7 to 6.1)
San Antón	48	9,373	5.1	5.2 (3.8 to 6.9)
San Miguel	28	9,511	2.9	2.5 (1.5 to 3.3)
San Basilio	17	5,509	3.1	3.0 (1.8 to 4.8)
Santiago Z	8	3,215	2.5	2.5 (1.1 to 4.9)
San Pio X	4	824	4.8	4.2 (1.1 to 10.8)

^a^SIR, standardized incidence ratio by neighborhood within Murcia city. Only results of neighborhoods with SIR >1 are represented; CI, confidence intervals.

*L. pneumophila* serogroup 1 was recovered from clinical samples of 19 patients; 18 samples were characterized. All were Pontiac (MAb 2+) Philadelphia MAb type and shared an identical molecular pattern by AFLP, PFGE-*Sfi*I, and AP-PCR.

### Case-Control Study

The descriptive study showed no common indoor source of exposure and determined that the outbreak was provoked by a common source located in the northern part of the city. The study hypothesis was that the outbreak had its origin in environmental contamination from cooling towers or other installations capable of producing and dispersing large quantities of aerosols potentially contaminated by legionellae.

A total of 85 cases and 170 controls were included in a case-control study. Participation in the case-control study was classified as recently proposed by Olsen et al. (*21*). The response rate among eligible cases was 89% (85/96) and 96% (85/89) among those eligible who were contacted. The response rate among persons selected as eligible controls was 51% (170/334) and 61% (170/279) among those eligible who were contacted.

The distribution of cases and controls according to sex, age, and residence was identical. No significant differences were found between cases and controls in any of the variables considered as a risk for or predisposing factor to the disease, as shown in Table 2. No differences were found with respect to education level or employment situation (Table 3). A strong association between visiting the city of Murcia and being ill with LD was found (OR 14.1, 95% CI 4.2 to 45.9).

**Table 2:** Risk factors for Legionnaires’ disease, Murcia, Spain

Predisposing factors	Cases n=85	Controls n=170
Smoking (%)	43.5	40.6
Alcohol intake (cc/week)	134	106
Chronic illness or immunosuppressive therapy (%)	16.5	14.7
Previous hospitalization (%)	1.2	1.8
Previous travel (%)	11.8	13.5

**Table 3:** Educational level and employment status for Legionnaires’ disease patients and controls, Murcia, Spain

Education and employment	Cases (n=85)	Controls (n=170)
**Educational level (%)**		
Primary	52	51.5
Secondary	38	37.3
University	8.2	11.2
**Employment status (%)**		
Employed	65.5	63.5
Unemployed	5.9	3.6
Retired	19.5	21.8
Housewife/husband	8.3	8.4
Student	1.2	3.l

The zone of exposure, defined either by the block surrounding hospital H or by a circle of 200 m in radius around hospital H, was significantly associated with illness in all eight models of multivariate analysis (Table 4). This zone of exposure also showed a much higher OR in every model. Thus, LD was 4.8–11.4 times more likely to develop in persons who passed through the zone around hospital H during the risk period than in persons who did not travel through this zone, independent of their having passed through the other zones. These results were also independent of the number of times the patient had visited the city.

**Table 4:** Association between Legionnaires’ disease and a patient’s traveling through specific areas of the northern part of the city of Murcia, Spain^a^

Area of city	Block-area study				Circle area study			
	Complete analysis		Restricted analysis		Complete analysis		Restricted analysis	
	OR (95% CI)	OR (95% CI)	OR (95% CI)	OR (95% CI)	OR (95% CI)	OR (95% CI)	OR (95% CI)	OR (95% CI)
Walking vs. not passing through	Walking vs. not passing through or passing by driving	Walking vs. not passing through	Walking vs. not passing through or passing by driving	Walking vs. not passing through	Walking vs. not passing through or passing by driving	Walking vs. not passing through	Walking vs. not passing through or passing by driving
Hospital H	10.2 (3.6 to 8.8)	9.7 (3.9 to 23.6)	10.7 (2.5 to 5.5)	6.0 (1.9 to 18.4)	6.9 (1.8 to 6.0)	6.4 (2.5 to 15.7)	11.4 (3.2 to 0.1)	4.8 (1.5 to 5.2)
Garden P	5.2 (1.0 to 5.8)	4.6 (1. to 17.0)						
Car-park X					5.1 (1.7 to 4.9)	3.6 (1.4 to 9.2)		
Commercial building						2.9 (1.1 to 7.4)		

^a^Areas associated to the disease in a multivariate analysis of data from eight different models. OR, odds ratio;

CI, confidence interval.

An association between the illness and walking through the zone was observed in the multivariate analysis for another three zones of exposure. However, none of these zones appeared in more than two of the eight models, and each had an OR that was lower or much lower than that for the zone around hospital H in the corresponding model.

### Nosocomial Outbreak at Hospital H

During the epidemiologic study of this community outbreak of LD, a nosocomial outbreak of LD at hospital H was discovered. In all 11 definite or probable nosocomial cases, some portion of the previous 10-day period of hospitalization coincided with the period when the cooling towers could have been active.

### Environmental Inspection and Microbiologic Study of Environmental Samples

**Figure 4:**
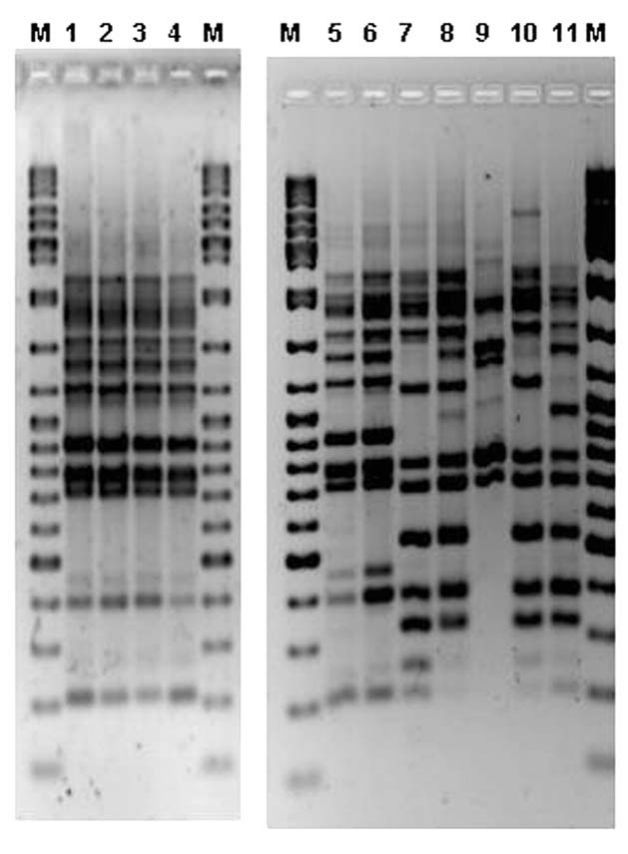
Amplified fragment length polymorphism (AFLP) gel containing outbreak human and environmental *Legionella pneumophila* serogroup 1 isolates. M, molecular weight marker (Ladder Mix, MBI Fermentas, UK). Lanes 1 and 2, two colonies from a cooling tower of the hospital H. Lines 3 and 4, human isolates. Lanes 5 and 6, human isolates. Lanes 7–11, different environmental isolates from several Murcia installations.

*L. pneumophila* was not recovered from water samples from the drinking water supply network in the city of Murcia. *L. pneumophila* serogroup 1 Pontiac (MAb 2+) was recovered from 22 installations (cooling towers of 11 buildings in the city and water storage tanks from 3 buildings). Ten of 11 cooling towers contained a Philadelphia MAb-type strain, but only two colonies, recovered in October from a cooling tower of hospital H, were indistinguishable from the patient strains by AFLP (Figure 4). Identical results were obtained when PFGE-*Sfi*I and AP-PCR were applied.

### Weather Conditions

Data provided by the Weather Centre of Murcia showed that during the last days of June and early July some atmospheric thermal inversion occurred every day, except one. Winds were predominantly from the northeast quadrant with a very low average speed (9 kph) and very high temperatures (33.5°C–35°C).

## Discussion

This LD outbreak is the largest to date in the world, with 449 confirmed cases and an estimated total number of cases of 650. The reported case-fatality rate (1%) is much lower than those observed in other community outbreaks (*22*,*23*). This rate can be attributed, at least partially, to the quick detection of the outbreak, early diagnosis of the disease, and appropriate treatment of patients. The explosive quality of the outbreak not only led patients to seek quick assistance at hospital emergency units but also helped clinicians to perform an accurate diagnosis and to immediately initiate adequate treatment, factors reported as linked to low case fatality (*24*,*25*). This explosive appearance could also be related to a lower presence of predisposing factors in case-patients in comparison with other community outbreaks (*7*,*9*,*13*), which could also partially explain the low case-fatality rate.

The investigation encountered obstacles, such as a large number of potential sources of environmental contamination located in the northern part of the city and the absence of environmental *Legionella* isolates identical to those of patients. The case-control study showed a significant association, with a high consistency between the analyzed models and with a high magnitude of association, between passing through the zone around hospital H and being ill with LD. Results were similar even when the area radius was expanded to 400 m. However, large overlap of areas was observed within this radius, and multicollinearity among zones was a common finding.

The case-control study was designed to select patients residing outside the city of Murcia. We decided on this approach for two reasons. First, the incidence of LD was almost 1% in some neighborhoods, a rate within the 0.1% to 5% attack rate described for this disease (*26*) Therefore, all the persons living in these quarters could possibly have been exposed to *Legionella*, as has been described in outbreaks of other transmissible diseases (*27*). If everyone had been exposed, finding incidence differences between persons exposed and those not exposed would have been almost impossible. Second, persons residing outside the city would probably have a more accurate memory of the itineraries they followed in Murcia some weeks previously and would probably have a lesser number of routes than persons living within the city. Conducting 255 personal interviews with questions about itineraries within 2 weeks from the last case and 4 weeks from the outbreak onset may also have been important to our findings.

One concern in case-control studies is that participation rate is not reported consistently (*21*). Indeed, this information is usually omitted in case-control studies of outbreaks, especially when controls are selected from a population database, as was our situation. A further complication was that the study had to be conducted in July, when many people go on holiday. In spite of achieving the participation of one in two controls whom we initially selected, we evaluated possible selection bias. We determined that it was unlikely to have occurred since neither socioeconomic status nor predisposing risk factors for LD differed significantly among cases and controls. Information bias overestimating this outcome was ruled out since news media did not mention hospital H among the probable sources of the outbreak.

Meteorologic conditions were favorable for the emission of aerosols to be dispersed in a horizontal manner. Low wind speed together with atmospheric thermal inversion between June 29 and July 1 would have facilitated the presence of the aerosols in the environment (*9*).

The result of the epidemiologic study was subsequently confirmed by the isolation of a strain retrieved on October 30 from a sample from one of the cooling towers of the same hospital; that strain is identical to the strain isolated from the patients. The difficulties found previously in the isolation of this strain were not unexpected. The day after the outbreak was detected, when the first sample was taken, the cooling towers of hospital H were highly chlorinated, which could explain why these first samples gave negative results. Later samples retrieved on four different dates between July 28 and September 13 showed positive results to *L. pneumophila* but were characterized as different strains from those from patients. This strain was only isolated upon the restarting of one tower after it was shut down for more than 1 month, a condition that favors the reappearance of *Legionella* (*8*,*12*,*13*). The fact that the same clone of *Legionella* can be found in an installation for long periods is also documented (*28*,*29*). The possible contamination of the tower by new *Legionella* from the water supply was ruled out since the strain linked to the outbreak was not found in samples collected from many other installations during the same period, including July to November.

The coincidence of a nosocomial LD outbreak in hospital H reinforces the previous hypothesis. A nosocomial outbreak of LD as part of a wider community outbreak of the disease has been described (*12*,*30*), although in other outbreaks originating in the cooling towers of a hospital no cases of nosocomial LD were identified (*7*). The use of double HEPA filters on air-intake vents in some hospitals could justify, at least in part, these contradictory observations.

Our research indicates that the cooling towers of a hospital located in the northeastern part of the city of Murcia were the origin of this community outbreak. This study underlines important risk factors that must be taken into account to prevent a new LD outbreak. First, cooling towers had to be identified by aerial and direct inspection in the absence of any census of such installations. Second, the size, location, and state of maintenance of cooling towers are very important. In contrast with epidemics associated with relatively small systems (*8*), this outbreak was related to a large refrigeration system that seems to have infected patients up to 1.3 km downwind to the west from the cooling tower; this finding suggests that airborne infection with *L. pneumophila* may extend over a large distance from the dissemination source, as has been reported elsewhere (*9*,*10*). Although most of the installations in the area showed inadequate maintenance, the cooling towers from hospital H were poorly maintained and had a high-risk size and location. Once the outbreak was identified, urgent measures were undertaken to clean, disinfect, or close possibly contaminated sources. The cooling tower that was the source of the outbreak was subsequently replaced by an air-cooled system.

Before June 2001, no specific national legislation existed in Spain concerning LD, although a recommendation guide and legislation existed in several Spanish autonomous regions that had had community LD outbreaks (*31*). As an immediate consequence of this outbreak, a national law about prevention and control of LD was enacted in Spain 20 days after the outbreak began (*32*). The extent of this outbreak is useful to assess the relative role of cooling towers as a source of LD and highlights the importance of prioritizing control measures related to cooling towers among strategies to prevent LD in the community. Compliance with these measures would help to reduce not only community outbreaks but also, perhaps, sporadic cases that could be due to infected cooling towers (*33*).
